# Supplementary material for: An open-source framework for large-scale, flexible evaluation of biomedical text mining systems
Source: J Biomed Discov Collab. 2008 Jan 29;3:1. doi: 10.1186/1747-5333-3-1 (PMC2276192; doi:10.1186/1747-5333-3-1)
Supplement: Additional file 1 — Evaluation platform source code. The additional file contains the software evaluation framework discussed in this paper. The most current release can be downloaded from SourceForge.net [1]. See the accompanying README file for instructions on installation and use. Also included in the distribution is a collection of UIMA wrappers for some commonly used BioNLP tools and annotated corpora. [file 1747-5333-3-1-S1.zip › bionlp_uima_repository-1.0.1/docs/api/src-html/edu/uchsc/ccp/uima/annotation/CCPTextAnnotation.html]

```
001    
002    
003    /* First created by JCasGen Tue Aug 28 16:44:10 MDT 2007 */
004    package edu.uchsc.ccp.uima.annotation;
005    
006    import org.apache.uima.jcas.JCas;
007    import org.apache.uima.jcas.JCasRegistry;
008    import org.apache.uima.jcas.cas.FSArray;
009    import org.apache.uima.jcas.cas.TOP_Type;
010    
011    import edu.uchsc.ccp.uima.mention.CCPClassMention;
012    
013    
014    /** The CCP TextAnnotation extends the base annotation class to include an annotation ID, the capability for multiple annotation spans, a link to the annotator responsible for generating the annotation, membership to annotation sets, and a link to a class mention which defines the class of this annotation.
015     * Updated by JCasGen Tue Aug 28 16:44:10 MDT 2007
016     * XML source: /home/williamb/eclipse/RELEASED_SOURCE_CODE/descriptors/typeSystem/CCPTypeSystem.xml
017     * @generated */
018    public class CCPTextAnnotation extends CCPAnnotation {
019      /** @generated
020       * @ordered 
021       */
022      public final static int typeIndexID = JCasRegistry.register(CCPTextAnnotation.class);
023      /** @generated
024       * @ordered 
025       */
026      public final static int type = typeIndexID;
027      /** @generated  */
028      public              int getTypeIndexID() {return typeIndexID;}
029     
030      /** Never called.  Disable default constructor
031       * @generated */
032      protected CCPTextAnnotation() {}
033        
034      /** Internal - constructor used by generator 
035       * @generated */
036      public CCPTextAnnotation(int addr, TOP_Type type) {
037        super(addr, type);
038        readObject();
039      }
040      
041      /** @generated */
042      public CCPTextAnnotation(JCas jcas) {
043        super(jcas);
044        readObject();   
045      } 
046    
047      /** @generated */  
048      public CCPTextAnnotation(JCas jcas, int begin, int end) {
049        super(jcas);
050        setBegin(begin);
051        setEnd(end);
052        readObject();
053      }   
054    
055      /** <!-- begin-user-doc -->
056        * Write your own initialization here
057        * <!-- end-user-doc -->
058      @generated modifiable */
059      private void readObject() {}
060         
061     
062        
063      //*--------------*
064      //* Feature: annotationID
065    
066      /** getter for annotationID - gets The annotation ID provides a means for identifying a particular annotation. Setting this ID is optional. The default value should be -1.
067       * @generated */
068      public int getAnnotationID() {
069        if (CCPTextAnnotation_Type.featOkTst && ((CCPTextAnnotation_Type)jcasType).casFeat_annotationID == null)
070          jcasType.jcas.throwFeatMissing("annotationID", "edu.uchsc.ccp.uima.annotation.CCPTextAnnotation");
071        return jcasType.ll_cas.ll_getIntValue(addr, ((CCPTextAnnotation_Type)jcasType).casFeatCode_annotationID);}
072        
073      /** setter for annotationID - sets The annotation ID provides a means for identifying a particular annotation. Setting this ID is optional. The default value should be -1. 
074       * @generated */
075      public void setAnnotationID(int v) {
076        if (CCPTextAnnotation_Type.featOkTst && ((CCPTextAnnotation_Type)jcasType).casFeat_annotationID == null)
077          jcasType.jcas.throwFeatMissing("annotationID", "edu.uchsc.ccp.uima.annotation.CCPTextAnnotation");
078        jcasType.ll_cas.ll_setIntValue(addr, ((CCPTextAnnotation_Type)jcasType).casFeatCode_annotationID, v);}    
079       
080        
081      //*--------------*
082      //* Feature: annotator
083    
084      /** getter for annotator - gets The annotator was responsible for generating this annotation.
085       * @generated */
086      public CCPAnnotator getAnnotator() {
087        if (CCPTextAnnotation_Type.featOkTst && ((CCPTextAnnotation_Type)jcasType).casFeat_annotator == null)
088          jcasType.jcas.throwFeatMissing("annotator", "edu.uchsc.ccp.uima.annotation.CCPTextAnnotation");
089        return (CCPAnnotator)(jcasType.ll_cas.ll_getFSForRef(jcasType.ll_cas.ll_getRefValue(addr, ((CCPTextAnnotation_Type)jcasType).casFeatCode_annotator)));}
090        
091      /** setter for annotator - sets The annotator was responsible for generating this annotation. 
092       * @generated */
093      public void setAnnotator(CCPAnnotator v) {
094        if (CCPTextAnnotation_Type.featOkTst && ((CCPTextAnnotation_Type)jcasType).casFeat_annotator == null)
095          jcasType.jcas.throwFeatMissing("annotator", "edu.uchsc.ccp.uima.annotation.CCPTextAnnotation");
096        jcasType.ll_cas.ll_setRefValue(addr, ((CCPTextAnnotation_Type)jcasType).casFeatCode_annotator, jcasType.ll_cas.ll_getFSRef(v));}    
097       
098        
099      //*--------------*
100      //* Feature: documentSectionID
101    
102      /** getter for documentSectionID - gets The document section ID is optionally used to log what section of a document this annotation is from. Values can be specified by the user. See edu.uchsc.ccp.util.nlp.document.DocumentSectionTypes for a few common sections.
103       * @generated */
104      public int getDocumentSectionID() {
105        if (CCPTextAnnotation_Type.featOkTst && ((CCPTextAnnotation_Type)jcasType).casFeat_documentSectionID == null)
106          jcasType.jcas.throwFeatMissing("documentSectionID", "edu.uchsc.ccp.uima.annotation.CCPTextAnnotation");
107        return jcasType.ll_cas.ll_getIntValue(addr, ((CCPTextAnnotation_Type)jcasType).casFeatCode_documentSectionID);}
108        
109      /** setter for documentSectionID - sets The document section ID is optionally used to log what section of a document this annotation is from. Values can be specified by the user. See edu.uchsc.ccp.util.nlp.document.DocumentSectionTypes for a few common sections. 
110       * @generated */
111      public void setDocumentSectionID(int v) {
112        if (CCPTextAnnotation_Type.featOkTst && ((CCPTextAnnotation_Type)jcasType).casFeat_documentSectionID == null)
113          jcasType.jcas.throwFeatMissing("documentSectionID", "edu.uchsc.ccp.uima.annotation.CCPTextAnnotation");
114        jcasType.ll_cas.ll_setIntValue(addr, ((CCPTextAnnotation_Type)jcasType).casFeatCode_documentSectionID, v);}    
115       
116        
117      //*--------------*
118      //* Feature: annotationSets
119    
120      /** getter for annotationSets - gets Annotation Sets provide an arbitrary means of categorizing and clustering annotations into groups.
121       * @generated */
122      public FSArray getAnnotationSets() {
123        if (CCPTextAnnotation_Type.featOkTst && ((CCPTextAnnotation_Type)jcasType).casFeat_annotationSets == null)
124          jcasType.jcas.throwFeatMissing("annotationSets", "edu.uchsc.ccp.uima.annotation.CCPTextAnnotation");
125        return (FSArray)(jcasType.ll_cas.ll_getFSForRef(jcasType.ll_cas.ll_getRefValue(addr, ((CCPTextAnnotation_Type)jcasType).casFeatCode_annotationSets)));}
126        
127      /** setter for annotationSets - sets Annotation Sets provide an arbitrary means of categorizing and clustering annotations into groups. 
128       * @generated */
129      public void setAnnotationSets(FSArray v) {
130        if (CCPTextAnnotation_Type.featOkTst && ((CCPTextAnnotation_Type)jcasType).casFeat_annotationSets == null)
131          jcasType.jcas.throwFeatMissing("annotationSets", "edu.uchsc.ccp.uima.annotation.CCPTextAnnotation");
132        jcasType.ll_cas.ll_setRefValue(addr, ((CCPTextAnnotation_Type)jcasType).casFeatCode_annotationSets, jcasType.ll_cas.ll_getFSRef(v));}    
133        
134      /** indexed getter for annotationSets - gets an indexed value - Annotation Sets provide an arbitrary means of categorizing and clustering annotations into groups.
135       * @generated */
136      public CCPAnnotationSet getAnnotationSets(int i) {
137        if (CCPTextAnnotation_Type.featOkTst && ((CCPTextAnnotation_Type)jcasType).casFeat_annotationSets == null)
138          jcasType.jcas.throwFeatMissing("annotationSets", "edu.uchsc.ccp.uima.annotation.CCPTextAnnotation");
139        jcasType.jcas.checkArrayBounds(jcasType.ll_cas.ll_getRefValue(addr, ((CCPTextAnnotation_Type)jcasType).casFeatCode_annotationSets), i);
140        return (CCPAnnotationSet)(jcasType.ll_cas.ll_getFSForRef(jcasType.ll_cas.ll_getRefArrayValue(jcasType.ll_cas.ll_getRefValue(addr, ((CCPTextAnnotation_Type)jcasType).casFeatCode_annotationSets), i)));}
141    
142      /** indexed setter for annotationSets - sets an indexed value - Annotation Sets provide an arbitrary means of categorizing and clustering annotations into groups.
143       * @generated */
144      public void setAnnotationSets(int i, CCPAnnotationSet v) { 
145        if (CCPTextAnnotation_Type.featOkTst && ((CCPTextAnnotation_Type)jcasType).casFeat_annotationSets == null)
146          jcasType.jcas.throwFeatMissing("annotationSets", "edu.uchsc.ccp.uima.annotation.CCPTextAnnotation");
147        jcasType.jcas.checkArrayBounds(jcasType.ll_cas.ll_getRefValue(addr, ((CCPTextAnnotation_Type)jcasType).casFeatCode_annotationSets), i);
148        jcasType.ll_cas.ll_setRefArrayValue(jcasType.ll_cas.ll_getRefValue(addr, ((CCPTextAnnotation_Type)jcasType).casFeatCode_annotationSets), i, jcasType.ll_cas.ll_getFSRef(v));}
149       
150        
151      //*--------------*
152      //* Feature: numberOfSpans
153    
154      /** getter for numberOfSpans - gets The number of spans comprising this annotation. The CCP TextAnnotation allows the use of multiple spans for a single annotation.
155       * @generated */
156      public int getNumberOfSpans() {
157        if (CCPTextAnnotation_Type.featOkTst && ((CCPTextAnnotation_Type)jcasType).casFeat_numberOfSpans == null)
158          jcasType.jcas.throwFeatMissing("numberOfSpans", "edu.uchsc.ccp.uima.annotation.CCPTextAnnotation");
159        return jcasType.ll_cas.ll_getIntValue(addr, ((CCPTextAnnotation_Type)jcasType).casFeatCode_numberOfSpans);}
160        
161      /** setter for numberOfSpans - sets The number of spans comprising this annotation. The CCP TextAnnotation allows the use of multiple spans for a single annotation. 
162       * @generated */
163      public void setNumberOfSpans(int v) {
164        if (CCPTextAnnotation_Type.featOkTst && ((CCPTextAnnotation_Type)jcasType).casFeat_numberOfSpans == null)
165          jcasType.jcas.throwFeatMissing("numberOfSpans", "edu.uchsc.ccp.uima.annotation.CCPTextAnnotation");
166        jcasType.ll_cas.ll_setIntValue(addr, ((CCPTextAnnotation_Type)jcasType).casFeatCode_numberOfSpans, v);}    
167       
168        
169      //*--------------*
170      //* Feature: spans
171    
172      /** getter for spans - gets This FSArray stores the CCPSpans which comprise this annotation. It should be noted that for an annotation with multiple spans, the default begin and end fields are set to the beginning of the first span and the end of the final span, respectively.
173       * @generated */
174      public FSArray getSpans() {
175        if (CCPTextAnnotation_Type.featOkTst && ((CCPTextAnnotation_Type)jcasType).casFeat_spans == null)
176          jcasType.jcas.throwFeatMissing("spans", "edu.uchsc.ccp.uima.annotation.CCPTextAnnotation");
177        return (FSArray)(jcasType.ll_cas.ll_getFSForRef(jcasType.ll_cas.ll_getRefValue(addr, ((CCPTextAnnotation_Type)jcasType).casFeatCode_spans)));}
178        
179      /** setter for spans - sets This FSArray stores the CCPSpans which comprise this annotation. It should be noted that for an annotation with multiple spans, the default begin and end fields are set to the beginning of the first span and the end of the final span, respectively. 
180       * @generated */
181      public void setSpans(FSArray v) {
182        if (CCPTextAnnotation_Type.featOkTst && ((CCPTextAnnotation_Type)jcasType).casFeat_spans == null)
183          jcasType.jcas.throwFeatMissing("spans", "edu.uchsc.ccp.uima.annotation.CCPTextAnnotation");
184        jcasType.ll_cas.ll_setRefValue(addr, ((CCPTextAnnotation_Type)jcasType).casFeatCode_spans, jcasType.ll_cas.ll_getFSRef(v));}    
185        
186      /** indexed getter for spans - gets an indexed value - This FSArray stores the CCPSpans which comprise this annotation. It should be noted that for an annotation with multiple spans, the default begin and end fields are set to the beginning of the first span and the end of the final span, respectively.
187       * @generated */
188      public CCPSpan getSpans(int i) {
189        if (CCPTextAnnotation_Type.featOkTst && ((CCPTextAnnotation_Type)jcasType).casFeat_spans == null)
190          jcasType.jcas.throwFeatMissing("spans", "edu.uchsc.ccp.uima.annotation.CCPTextAnnotation");
191        jcasType.jcas.checkArrayBounds(jcasType.ll_cas.ll_getRefValue(addr, ((CCPTextAnnotation_Type)jcasType).casFeatCode_spans), i);
192        return (CCPSpan)(jcasType.ll_cas.ll_getFSForRef(jcasType.ll_cas.ll_getRefArrayValue(jcasType.ll_cas.ll_getRefValue(addr, ((CCPTextAnnotation_Type)jcasType).casFeatCode_spans), i)));}
193    
194      /** indexed setter for spans - sets an indexed value - This FSArray stores the CCPSpans which comprise this annotation. It should be noted that for an annotation with multiple spans, the default begin and end fields are set to the beginning of the first span and the end of the final span, respectively.
195       * @generated */
196      public void setSpans(int i, CCPSpan v) { 
197        if (CCPTextAnnotation_Type.featOkTst && ((CCPTextAnnotation_Type)jcasType).casFeat_spans == null)
198          jcasType.jcas.throwFeatMissing("spans", "edu.uchsc.ccp.uima.annotation.CCPTextAnnotation");
199        jcasType.jcas.checkArrayBounds(jcasType.ll_cas.ll_getRefValue(addr, ((CCPTextAnnotation_Type)jcasType).casFeatCode_spans), i);
200        jcasType.ll_cas.ll_setRefArrayValue(jcasType.ll_cas.ll_getRefValue(addr, ((CCPTextAnnotation_Type)jcasType).casFeatCode_spans), i, jcasType.ll_cas.ll_getFSRef(v));}
201       
202        
203      //*--------------*
204      //* Feature: classMention
205    
206      /** getter for classMention - gets The CCP ClassMention indicates the type (or class) for this annotation.
207       * @generated */
208      public CCPClassMention getClassMention() {
209        if (CCPTextAnnotation_Type.featOkTst && ((CCPTextAnnotation_Type)jcasType).casFeat_classMention == null)
210          jcasType.jcas.throwFeatMissing("classMention", "edu.uchsc.ccp.uima.annotation.CCPTextAnnotation");
211        return (CCPClassMention)(jcasType.ll_cas.ll_getFSForRef(jcasType.ll_cas.ll_getRefValue(addr, ((CCPTextAnnotation_Type)jcasType).casFeatCode_classMention)));}
212        
213      /** setter for classMention - sets The CCP ClassMention indicates the type (or class) for this annotation. 
214       * @generated */
215      public void setClassMention(CCPClassMention v) {
216        if (CCPTextAnnotation_Type.featOkTst && ((CCPTextAnnotation_Type)jcasType).casFeat_classMention == null)
217          jcasType.jcas.throwFeatMissing("classMention", "edu.uchsc.ccp.uima.annotation.CCPTextAnnotation");
218        jcasType.ll_cas.ll_setRefValue(addr, ((CCPTextAnnotation_Type)jcasType).casFeatCode_classMention, jcasType.ll_cas.ll_getFSRef(v));}    
219      }
220    
221
```
